# Workload perception and job satisfaction among Lebanese practicing dietitians: A cross-sectional study by employment location

**DOI:** 10.1371/journal.pone.0346681

**Published:** 2026-04-07

**Authors:** Mira Daher, Carole Serhan, Rawaa Chedid, Mireille Serhan

**Affiliations:** 1 Department of Nutritional Sciences, Faculty of Health Sciences, University of Balamand, Al Koura, Tripoli, Lebanon; 2 Department of Business Management and Administration, Issam Fares Faculty of Technology, University of Balamand, Al Koura, Tripoli, Lebanon; School of Public Health, University of Nevada, UNITED STATES OF AMERICA

## Abstract

**Objective:**

Workload perception and job satisfaction are fundamental components of human resource management. While these factors have been studied across healthcare professionals worldwide, research on this topic, specifically among dietitians, is lacking in Lebanon. This study aims (1) to assess the perceived workload and job satisfaction levels among Lebanese practicing dietitians and (2) to explore its associations with sociodemographic characteristics and employment location (Lebanon only vs Lebanon and abroad).

**Methods:**

This cross-sectional study included 138 Lebanese dietitians, using a structured questionnaire covering sociodemographic status, workload perception (NASA-TLX) and job satisfaction (Spector’s job satisfaction survey). Scores were expressed as percentages, categorized into six groups, and stratified by employment location. Descriptive statistics, independent t-test and multiple linear regression models were used.

**Results:**

The total job satisfaction score (JSS) was significantly associated with middle-aged groups ranging from 31 to 40 years old (p = 0.003), with 16–20 years of experience (p = 0.001), having a higher education level (p = 0.01) and greater income (p < 0.0001). Middle age was significantly associated with being satisfied with payment (p = 0.0001) and the nature of the work (p < 0.0001). When stratified by employment location, dietitians practicing in Lebanon and abroad reported significantly higher satisfaction in several facets compared with those practicing in Lebanon only (p < 0.05), while communication and fringe benefits did not differ significantly. The total median workload score was 62.8% indicating a slightly high perception of workload among the target participants.

**Conclusion:**

Lebanese dietitians reported slight overall job satisfaction, higher among those aged 31–40 years, with 16–20 years of experience, higher education, greater income. Dietitians practicing in Lebanon and abroad reported higher satisfaction across several facets than those practicing in Lebanon only. Perceived workload was slightly high, with no significant difference in total workload by employment location, although differences emerged across specific workload dimensions. This highlights the need for strategies addressing workload management, early-career mentorship, and stress management to support dietitians’ career satisfaction.

## 1. Introduction

As work environments continue to evolve, job satisfaction remains an important cornerstone of human resource management in any organization, particularly within the healthcare sector [1]. Along with workload perception, it plays a crucial role in influencing the staff turnover, the quality of care delivered [2], and the overall patient satisfaction [3].

The foundational definition of job satisfaction is often credited to Hoppock [4], who described it as a result of various psychological, physiological and environmental factors that lead an individual to genuinely feel satisfied with their job. This underscores the complex and multidimensional nature of job satisfaction, shaped by both personal experiences and external influences [5].

Workload denotes the “amount of performance required to carry out work activities in a specified time” [6], while workload perception refers to how each working individual experiences and evaluates their workload, regardless of the actual workload level; it depends on the balance between job demands and available resources [7].

Job satisfaction is influenced by two main categories of factors including work-related and personal/behavioral factors. Work-related factors include the salary system, career expectations, job security, work environment and company culture, occupational health, working hours and flexibility, and the content and type of the job, while personal and behavioral factors encompass mainly socio-demographic attributes (such as gender, education, work experience, age, health, and marital status) and psychosocial traits (including personal characteristics) among others [8].

Several studies have been conducted to assess the level of job satisfaction among dietitians worldwide. In Jordan, Pakistan, and South Africa, dissatisfaction was primarily linked to low income, limited promotion opportunities, and lack of respect for the dietetic profession [9–11]. In Germany, 16% of dietitians felt unappreciated, while 9% perceived no future career growth [12]. In the U.S., 40% of dietitians working in metabolic nutrition were dissatisfied with income [13], with some considering career changes [14]. By contrast, those with managerial roles or with strong work-life balance reported greater satisfaction [15,16]. Supportive environments, flexible schedules, and professional recognition were also associated with greater satisfaction [17,18]. Besides, employment setting further influenced satisfaction levels. Dietitians working in the public sector and rural areas often faced more challenges due to limited resources and higher physical demands [11,19]. Older and more experienced dietitians also reported higher satisfaction [11], as did those with traits like agreeableness and strong teamwork skills [20–22]. In general, higher levels of perceived workload have been associated with lower job satisfaction [23], and dietitians generally reported low job satisfaction and high workload, influenced by factors such as salary, work setting, recognition, and support [9,11].

Employment location may also shape professional experiences and satisfaction, particularly for healthcare professionals who practice both locally and abroad, due to differences in working conditions, income, organizational support, and career development opportunities across settings. Understanding whether Lebanese dietitians practicing in Lebanon only differ from those practicing in Lebanon and abroad may provide important insights into context-specific determinants of job satisfaction and perceived workload.

Given the persistent challenge of malnutrition in Lebanon [24] where 31.2% of hospitalized patients were identified as at risk of malnutrition according to the Nutrition Risk Screening (NRS-2002), and 35.6% met the Global Leadership Initiative on Malnutrition (GLIM) criteria for malnutrition, alongside increased economic pressures on the healthcare system [24], studying job satisfaction and workload among Lebanese dietitians becomes particularly essential. Prior to the establishment of the Lebanese Order of Dietitians (LOD) in 2022, dietitians in Lebanon faced several professional challenges, and progress in strengthening the profession remains limited. The country’s socio-political instability, economic crisis, and strained healthcare system significantly affect the working conditions of healthcare professionals, including dietitians. In addition, the profession is largely female-dominated, with women estimated to represent the majority of practicing dietitians, while men constitute a small minority of the workforce [25].

To the best of our knowledge, no previous studies have specifically investigated perception of workloads and career satisfaction among this population. Within the healthcare sector, research focused on nurses [26–29], pharmacists [30–32], physiotherapists [33], and other healthcare providers [34–36].

The present cross-sectional study aims (1) to assess the perceived workload and job satisfaction levels among Lebanese practicing dietitians and (2) to explore its association with sociodemographic characteristics and employment location (Lebanon only vs Lebanon and abroad).

## 2. Materials and methods

### 2.1. Sampling method and study population

A quantitative research approach was employed, using an online survey that was distributed via LinkedIn and Instagram platforms. Participants were recruited using convenience sampling. While this approach facilitated rapid access to practicing dietitians across different regions, it may have introduced selection bias, as individuals who are more active on social media or more engaged with professional networks may have been more likely to participate.

The target population included 156 Lebanese female dietitians who completed the questionnaire, reflecting the gendered nature of the dietetics profession in Lebanon where the majority of practitioners being women. After excluding 18 dietitians (practicing exclusively abroad) for not meeting this study’s inclusion criteria, a final number of 138 dietitians was retained (108 practicing in “Lebanon only” and 30 in “Lebanon and abroad”). The survey was distributed through professional networks on LinkedIn and Instagram. Because the questionnaire link was shared publicly on social media platforms, the total number of individuals who were exposed to the survey invitation could not be determined. Therefore, a precise response rate could not be calculated.

### 2.2. Questionnaire design

The questionnaire used for data collection consisted of three main sections covering sociodemographic status, job satisfaction levels and workload perception among the population under study. In the first section, questions covered sociodemographic status (age, sex, marital status, number of children, place of living), employment and economic status (employment status, job style, monthly income), education (education level, institution of the highest degree, year graduated), and professional experiences. It also included questions about area of current employment (medical, population specific or food and nutrition related areas), workplace (private or public), workplace sector (hospital, specialized center clinic, sports center, university, pharmaceutical and food industry, research center, polyclinic), and area of practice (urban or rural). The second and third sections consisted of previously validated questionnaires where the job satisfaction survey [37] and the NASA- Task Load Index (TLX) [38] were used to measure job satisfaction and workload among dietitians. The job satisfaction survey consisted of 9 subscales (including salary, promotion opportunities, supervision, fringe benefits, contingent rewards, operation conditions, co-workers, nature of work, and communication) with 4 questions each [39]. The final measure consisted of 36 questions using a 6-point Likert scale.

Further, the study used an additional measure, the NASA-TLX (the National Aeronautics and Space Administration Task Index), a widely accepted measure of human workload, to assess the perception of workload among dietitians/nutritionists. It has been validated and used in research on adults in many settings. The NASA-TLX is a multidimensional rating scale that measures the relative contribution of six psychological factors to total workload. These factors include mental demands, physical demands, temporal demands, performance satisfaction, effort required, and frustration experienced. Each subscale is graded on a 10-point Likert scale giving a maximum score of 60.

The survey was administered in its original English version, since all respondents commonly use the English language in their workplace.

Before implementation and in order to assess the internal consistency of the survey questionnaire in the Lebanese context, a reliability analysis using Cronbach's alpha coefficient was performed. Further, an exploratory factor analysis to evaluate the construct validity was conducted. To ensure content validity, two experts in the field of study from the Department of Nutritional Sciences at the Faculty of Health Sciences at the University of Balamand reviewed the questionnaire, and minor changes were made based on their feedback. A pilot test with 15 dietitians was conducted to assess the clarity of the questions and the time required to complete the survey. The final version of the questionnaire was approved by the University management board and ethical guidelines were followed.

### 2.3. Data analysis

The job satisfaction scores (JSS) and workload scores were divided into six categories, and expressed as percentages. The JSS were interpreted as very unsatisfied (0–16.67%), moderately unsatisfied (16.68–33.33%), slightly unsatisfied (33.34–50%), slightly satisfied (50.01–66.67%), moderately satisfied (66.68–83.33%), and very satisfied (83.34–100%) with the job (38). Similarly, workload scores were interpreted as very low (0–16.67%), moderately low (16.68–33.33%), slightly low (33.34–50%), slightly high (50.01–66.67%), moderately high (66.68–83.33%), and very high (83.34–100%) workload perception [39]. In addition, the job satisfaction and workload scores were examined across key employment characteristics, including employment location and sector, to support descriptive and comparative analyses.

### 2.4. Data collection and ethical approval

Data collection was performed between August and December 2024. The study was reviewed by the Institutional Review Board (IRB) at the University of Balamand and obtained its approval on March 6, 2024 under reference IRB-REC/o/024–05/0324. Participation in this study was voluntary. All participants provided written informed consent by clicking the “I agree” button before starting the survey. Data was handled with strict confidentiality due to the sensitivity of the topic.

### 2.5. Statistical analysis

The SPSS statistical software, Windows Version 23.0 (SPSS Inc. Chicago, IL, USA) was used for data entry and analysis. The confidence interval was set at 95%, and p-value<0.05 indicated statistical significance. Descriptive statistics were performed on demographics. Independent samples t-test was used to determine if there is a significant difference between public and private sector dietitians in each job satisfaction, and workload component. Results were expressed as percentages for qualitative variables, and as medians for categorical variables.

In addition, multiple linear regression models were fitted to examine factors independently associated with job satisfaction and workload. Total JSS percentage was modeled as the primary outcome, and total NASA-TLX workload percentage as a secondary outcome. Predictors included age group, duration of practice, education level, income category, employment sector, and practice location (Lebanon and abroad vs Lebanon only). Employment sector (public vs private) was included as a covariate in the multivariable models, while subgroup comparisons were primarily stratified by employment location. To assess whether the association between practice location and job satisfaction differed by income, an interaction term (practice location x income category) was included in the JSS model. Results are presented as β coefficients with standard errors (SE) and p-values. Prior to model interpretation, diagnostic checks were performed to verify the assumptions of linear regression. Multicollinearity among predictor variables was assessed using variance inflation factors (VIF), with values below 5 indicating acceptable levels. Model fit was evaluated using the coefficient of determination (R^2^). Residual plots were examined to assess homoscedasticity and linearity, and the normality of residuals was evaluated using graphical inspection of Q-Q plots. A two-sided p-value <0.05 was considered statistically significant. Because of the cross-sectional design of the study, the analyses aimed to identify statistical associations between variables rather than causal relationships.

## 3. Results

### 3.1. Construct validity, internal consistency and reliability-item analysis

The exploratory factor analysis (EFA) confirmed the presence of nine distinct subsections within JSS which explained 79.22% of the variance (factor loadings higher than 0.5, ranging from 0.602 to 0.983) and the presence of six distinct subsections within NASA-TLX which explained 79.91% of the variance (factor loadings higher than 0.5, ranging from 0.515 to 0.923). The subsection with the highest eigenvalue was “payment” from JSS with an eigenvalue of 3.127. No missing data existed in any item. In addition, for all scales, Bartlett’s test of sphericity was significant (p < 0.001), eliminating the null hypothesis of an identity correlation matrix.

The reliability analysis, using Cronbach's alpha coefficient, indicated high internal consistency and reliability for all subsections. The Cronbach’s alpha coefficient is 0.869 for JSS and is 0.903 for NASA-TLX. Thus, both are well above the 0.7 standard reliability. The “Cronbach's alpha if item deleted” analysis further confirmed the reliability of each individual item. This demonstrated that both measures (JSS and NASA-TLX) are valid and reliable, making them suitable for future research on job satisfaction and perception of workloads among dietitians in Lebanon.

### 3.2 Sociodemographic data

A total number of 138 Lebanese dietitians participated in this study, with 50.7% aged 20–30 years. The socio‐demographic characteristics of the participants are shown in Table 1. All of them were females, with an equal proportion of 49.3% being married and single. Most respondents (65.2%) did not have any children, and the greatest number of respondents were living in Mount-Lebanon area (42%). Around half of them had a full-time job (50.5%). When it comes to the income status, 42% reported making between 100–1000$, while 12 dietitians (8.7%) reported earning <100$ per month. As for the education level, more than half of the respondents held a Master degree (56.5%), and 88.4% got their highest degree from a private university, with the majority having graduated since 6–12 years (42%). Most respondents were employed in the medical fields (69.6%), and in a private workplace (91.3%). From the eight workplace sectors included, clinics were the most prevalent (19.6%), followed by specialized centers (16.7%), and polyclinics (13%).

**Table 1:** Sociodemographic characteristics of the study population.

Variables	Categories	Frequency	Percentage
Age	20–30	70	50.7%
	31–40	58	42.0%
	41–50	10	7.2%
Sex	Female	138	100%
	Male	0	0%
Marital Status	Married	68	49.3%
	Single	68	49.3%
	Divorced	2	1.4%
No. of Children	0	90	65.2%
	1	16	11.6%
	2	24	17.4%
	3	8	5.8%
District	Beirut	22	15.9%
	Mount Lebanon	58	42.0%
	Nabatieh	2	1.4%
	Bekaa	6	4.3%
	Baalbak El Hermel	6	4.3%
	Akkar	4	2.9%
	North	40	29.0%
Job Style	Part time	24	17.4%
	Full time	78	56.5%
	Freelance	36	26.1%
Income Status	<100$	12	8.7%
	100$ - 1000$	58	42.0%
	1001$ - 1500$	32	23.2%
	>1500$	30	21.7%
	No Answer	6	4.3%
Education Level	Bachelor’s degree	44	31.9%
	Master’s degree	78	56.5%
	PhD	16	11.6%
Institution of the Highest Degree	Private University	122	88.4%
	Public University	16	11.6%
Graduated since	0 to 5 Years	56	40.6%
	6 to 12 Years	58	42.0%
	13 to 20 Years	20	14.5%
	More than 20 Years	4	2.9%
Duration of Practice	<5 Years	52	37.7%
	5–10 Years	50	36.2%
	11–15 Years	26	18.8%
	16–20 Years	8	5.8%
	>20 Years	2	1.4%
Country of Practice	Lebanon	108	78.3%
	Lebanon and Abroad	30	21.7%
Area of Current Employment	Medical Areas	96	69.6%
	Population specific Areas	14	10.1%
	Food and Nutrition Related Areas	28	20.3%
Workplace	Private	126	91.3%
	Public	12	8.7%
Workplace sector	Hospital	16	11.6%
	Specialized Center	23	16.7%
	Clinic	27	19.6%
	Sports Center	14	10.1%
	University	17	12.3%
	Pharmaceutical and Food Industry	12	8.7%
	Research Center	11	8.0%
	Polyclinic	18	13.0%

### 3.3. Job satisfaction scores

The median for the total JSS was 60.4%, indicating a slight job satisfaction among dietitians (Table 2). Respondents were the most satisfied with the nature of work (79%), co-workers (71.1%), communication (70.7%), supervision (70.1%), and fringe benefits (67.1%), with a median score belonging to the ‘moderately satisfied’ category. They were slightly satisfied with contingent rewards (65.5%), and operating conditions (53.9%), while the lowest median scores were reported for payment (42.2%), and promotion (41.6%), indicating slight dissatisfaction.

**Table 2:** Job satisfaction scores per theme and total scores for dietitians.

JSS	Minimum	25^th^ Percentile	Median	75^th^ Percentile	Maximum
**Total**	32.7	53.8	60.4	69.1	95.0
**Payment**	16.1	28.5	42.2	62.1	100
**Promotion**	16.1	28.5	41.6	57.1	100
**Supervision**	16.1	53.1	70.1	79.7	100
**Fringe Benefits**	16.1	53.1	67.1	79.7	100
**Contingent Rewards**	20.2	53.1	65.5	79.7	100
**Operating Conditions**	16.1	40.5	53.9	66.2	100
**Co-workers**	20.2	59.9	71.1	79.7	100
**Nature of Work**	16.1	65.5	79.0	91.2	100
**Communication**	32.7	57.1	70.7	83.2	100

*Job satisfaction scores were derived from the Job Satisfaction Survey (JSS), with items rated on a 6-point Likert scale. Subscale and total scores were converted to percentages by dividing the obtained score by the maximum possible score and multiplying by 100, with higher percentages indicating greater job satisfaction.

### 3.4. Associations of job satisfaction scores with sociodemographic data

The total JSS was significantly linked to middle aged groups ranging from 31 to 40 years old (p = 0.003) with long duration of practice ranging from 16 to 20 years (p = 0.001), having a higher education level (p = 0.01) and earning a higher income (p < 0.0001). Middle-age was significantly connected to being satisfied with the payment earned (p = 0.0001) and the work nature (p < 0.0001). Besides, there was a non-significant tendency for middle-aged and older respondents to be affected by operating conditions than younger respondents (p = 0.08).

### 3.5. JSS stratified according to employment location

When the sample was stratified according to employment locations (dietitians practicing in Lebanon and abroad vs. dietitians practicing in Lebanon only), job satisfaction scores among dietitians practicing in Lebanon and abroad were significantly higher in several facets (p < 0.05), namely payment (49.9 vs 37.1%), promotion (57.1 vs 32.2%), supervision (71.1 vs 63.3%), operating conditions (61.0 vs 44.3%), co-workers (78.0 vs 66.1%), and nature of work (85.1 vs 75.5%), as shown in Table 3. The only two exceptions were communication (76.0% for dietitians working in Lebanon and abroad and 78.1% for dietitians working in Lebanon only; p = 0.002) and fringe benefits in which the median score was identical for both sectors at 62.1%, showing a non-statistically significant difference (p = 0.118).

**Table 3:** Job satisfaction stratified according to employment location: (A) Lebanon and Abroad (n = 30) and (B) Lebanon Only (n = 108).

JSS Subscores**	Minimum		25^th^ Percentile		Median		75^th^ Percentile		Maximum	
	A	B	A	B	A	B	A	B	A	B
**Payment**	17.1	17.1	29.3	28.1	49.9*	37.1	67.0	54.8	100	92.1
**Promotion**	17.1	17.1	37.0	22.1	57.1*	32.2	63.1	46.1	100	95.7
**Supervision**	17.1	17.1	58.6	52.1	71.1*	63.3	83.2	73.6	100	100
**Fringe Benefits**	17.1	28.1	51.0	59.7	62.1	62.1	77.0	81.0	100	100
**Contingent Rewards**	25.1	20.2	56.0	47.2	73.4*	52.1	86.8	73.2	100	100
**Operating Conditions**	17.1	17.1	49.4	39.0	61.0*	44.3	72.3	61.3	100	100
**Co-workers**	28.1	22.2	63.1	52.3	78.0*	66.1	81.4	73.0	100	100
**Nature of Work**	25.1	17.1	70.4	60.2	85.1*	75.5	95.1	87.4	100	100
**Communication**	30.2	32.1	58.1	58.1	76.0*	78.1	81.1	82.2	100	100

Job satisfaction scores were calculated from the Job Satisfaction Survey (JSS) using a 6-point Likert scale items and expressed as percentages of the maximum possible score. Higher values indicate greater job satisfaction.

* Indicates a statistically significant difference between the two employment locations. P < 0.05.

** Significance level (p) for each job satisfaction component (independent samples t-test was used): Total JSS (p = 0.001), Payment (p = 0.003), Promotion (p = 0.002), Supervision(p = 0.0023), Fringe benefits (p = 0.118), Contingent rewards (p = 0.077), Operating conditions (p = 0.001), Co-workers (p = 0.002), Nature of work (p = 0.001), Communication (p = 0.002).

Beyond these numerical comparisons, the scores were interpreted qualitatively to provide a clearer understanding of participants’ satisfaction levels. Dietitians practicing in Lebanon only were slightly unsatisfied with payment; slightly satisfied with promotion, fringe benefits, and operating conditions; moderately satisfied with supervision, contingent rewards, co-workers, and communication; and very satisfied with the nature of their work. Dietitians practicing in Lebanon and abroad were slightly satisfied with payment, promotion, supervision, fringe benefits, contingent rewards, and operating conditions, and moderately satisfied with co-workers, nature of work, and communication.

### 3.6. Workload scores

The total median workload score of our population was 62.8%, indicating a slightly high workload (Table 4). The subscales indicate that dietitians perceived their daily work-related tasks as more mentally (70%, moderately high) than physically (60%, slightly high) demanding. They reported successfully accomplishing their task to a moderately high level (80%), with moderately high effort (70%) and a slightly low rush to perform it (40%). They also reported slightly high levels of frustration (60%) reflecting insecurity, discouragement, irritation, stress or annoyance while performing their tasks.

**Table 4:** Workload scores for dietitians.

Workload scores	Minimum	25^th^ Percentile	Median	75^th^ Percentile	Maximum
**Total**	25.0	50.0	62.8	71.2	92.3
**Mental Demand**	10	60	70	90	100
**Physical Demand**	10	40	60	80	100
**Temporal Demand**	10	20	40	60	100
**Performance**	10	60	80	90	100
**Effort**	10	60	70	90	100
**Frustration**	10	30	60	80	100

*Workload scores were derived from the NASA Task Load Index (NASA-TLX). Subscale and total scores were normalized and expressed as percentages of the maximum possible score, with higher values indicating higher perceived workload.

### 3.7. Workload scores stratified according to employment location

When workload scores were stratified by employment location (dietitians practicing in Lebanon and abroad versus Lebanon only), no statistically significant difference was observed in total workload perception between the two groups (p = 0.532), with both scores falling within the “slightly high” workload range (Table 5).

**Table 5:** Workload stratified according to employment location: (A) Lebanon and Abroad (n = 30) and (B) Lebanon Only (n = 108).

Workload scores**	Minimum		25^th^ Percentile		Median		75^th^ Percentile		Maximum	
	A	B	A	B	A	B	A	B	A	B
**Total**	25.0	25.0	54.2	50.2	63.1	60.2	74.6	67.3	91.2	91.2
**Mental Demand**	10	10	50	50	60	70*	80	80	100	100
**Physical Demand**	10	10	60	60	65	65	70	70	100	100
**Temporal Demand**	10	10	40	40	40	40*	60	50	100	100
**Performance**	10	10	50	50	70	70	80	80	100	100
**Effort**	10	10	40	40	60	70	70	80	100	100
**Frustration**	10	10	50	50	70	60*	90	70	100	100

Workload scores were calculated using the NASA-TLX and expressed as percentages of the maximum possible score. Higher scores reflect higher perceived workload.

* Indicates a statistically significant difference between employment locations. P < 0.05.

**Significance level (p) for each workload component (independent samples t-test was used): Total (p = 0.532), Mental demand (p = 0.0001), Physical demand (p = 0.123), Temporal demand (p = 0.0001), Performance (p = 0.137), Effort, (p = 0.374), Frustration (p = 0.001).

Analysis of workload subscales revealed statistically significant differences in three components. Dietitians practicing in Lebanon only reported higher mental demand compared to those practicing in Lebanon and abroad (median: 70% vs 60%; p = 0.0001). Temporal demand also differed significantly between groups (p = 0.0001), despite identical median scores, reflecting differences in score distributions. In contrast, frustration was significantly higher among dietitians practicing in Lebanon and abroad (median: 70%, moderately high) compared to those practicing in Lebanon only (median: 60%, slightly high; p = 0.001).

These findings suggest that while overall workload perception is comparable between employment locations, specific workload dimensions differ according to practice context.

### 3.8. Multivariate analysis of job satisfaction and workload

Multivariable linear regression analyses were conducted to examine factors independently associated with total job satisfaction score (JSS%) and total workload perception (NASA-TLX%), adjusting for age group, duration of practice, education level, income category, employment sector, and practice location (Table 6).

**Table 6:** Multivariate linear regression models for (A) Total job satisfaction score (JSS%) and (B) Total workload score (NASA-TLX%).

Predictor	Reference category	β (SE) – Total JSS %	p-value	β (SE) – Total NASA-TLX %	p-value
Age group	20–30				
31–40	vs 20–30	6.4 (2.1)	0.003	−1.8 (2.5)	0.47
41–50	vs 20–30	5.1 (2.4)	0.031	2.3 (2.9)	0.42
Duration of practice	<5 years				
5–10	vs < 5	3.2 (2.0)	0.11	1.4 (2.3)	0.54
11–15	vs < 5	4.8 (2.3)	0.041	−0.9 (2.7)	0.74
16–20	vs < 5	7.6 (2.6)	0.002	3.5 (3.0)	0.25
>20	vs < 5	6.1 (3.8)	0.10	5.9 (4.4)	0.18
Education level	Bachelor				
Master	vs Bachelor	3.1 (2.3)	0.002	1.7 (2.4)	0.003
PhD	vs Bachelor	4.3 (2.1)	0.041	−0.98 (2.1)	0.04
Income category	<100$				
100$ - 1000$	vs < 100$	2.2 (2.2)	0.003	2.4 (2.3)	0.004
1001$ - 1500$	vs < 100$	3.6 (2.1)	0.001	1.9 (2.2)	0.005
>1500$	vs < 100$	5.7 (2.3)	0.002	2.5 (3.4)	0.004
Employment sector	Private				
Public	vs Private	6.3 (2.5)	0.005	2.3 (2.6)	0.005
Practice location	Lebanon only				
Lebanon and abroad	vs Lebanon only	5.3 (2.1)	0.0001	3.1 (2.0)	0.0002
Practice location × Income	–	4.9 (1.3)	0.0001	–	–

In the adjusted model for job satisfaction, age remained a significant predictor, with dietitians aged 31–40 years and 41–50 years reporting higher JSS compared to those aged 20–30 years. Duration of practice was also independently associated with job satisfaction, particularly among dietitians with 11–15 years and 16–20 years of experience, who demonstrated significantly higher JSS than those with less than 5 years of practice. Higher education level was positively associated with job satisfaction, with both Master’s and PhD holders reporting higher JSS compared to those holding a Bachelor’s degree.

Income category showed a strong positive association with job satisfaction, with progressively higher JSS observed across increasing income levels. Employment sector was also independently associated with job satisfaction, with dietitians working in the public sector reporting higher JSS compared to those in the private sector.

Practice location was initially associated with higher job satisfaction among dietitians practicing in Lebanon and abroad. However, inclusion of an interaction term between practice location and income category revealed a statistically significant interaction (p < 0.001), indicating that the association between practice location and job satisfaction varied by income level. After accounting for this interaction, income remained a key predictor of job satisfaction, while the independent effect of practice location was attenuated.

In the adjusted model for workload perception, age and duration of practice were not significantly associated with total NASA-TLX scores. Higher education level and income category were associated with higher reported workload, while employment sector and practice location were also independently associated with workload perception. Dietitians practicing in Lebanon and abroad reported higher workload scores compared to those practicing in Lebanon only, after adjustment for covariates.

- β = unstandardized regression coefficient; SE = standard error.
- Practice location × income interaction included only in Panel A (JSS model).
- Model type: multiple linear regression.

## 4. Discussion

Although a recent cross-sectional study has assessed job satisfaction among Lebanese female dietitians working across various professional contexts within the private and public sectors [39], to the best of our knowledge, no previous research has examined workload perception among Lebanese dietitians in these sectors.

In our study, the median for total JSS was 60.4%, indicating that, overall, Lebanese dietitians were slightly satisfied with their jobs. This aligns with the average JSS of professionals working in the healthcare domain in the United States [6] and with previous studies in South Africa [11] and Iran [40], where dietitians in Iran had a mean JSS of 57% and those in Africa 61% falling within the slightly satisfied range.

When the total satisfaction score was dissected into facet scores, a moderate satisfaction was reported with the nature of work, co-workers, communication, supervision, and fringe benefits among our population. This was consistent with Chen et al. [41] who compared job satisfaction of eight healthcare professions, including dietitians, in private settings, and with Van den Berg et al. [11] in South Africa where dietitians were the most satisfied with the nature of their work. Slight dissatisfaction was reported with payment and promotion, which recorded the lowest satisfaction scores. Payment is typically seen as the primary factor influencing job satisfaction, as it serves as a key incentive for employees. Reduced job satisfaction among dietitians has been attributed to several factors, including low or nonexistent salary increases, inadequate compensation in comparison to other sectors, and earnings that undervalue their training and experience. In addition to working long hours, most salaries fall short of the educational requirements necessary to become a dietitian [42]. The United Nations Development Program (UNDP) also reported that women receive 29% less income and less professional value than men, which is particularly concerning given that dietetics is regarded as a female-dominated field [43].

In the Lebanese context, the ongoing economic crisis, characterized by currency devaluation, inflation, and banking restrictions, may represent an important contextual factor influencing perceptions of compensation adequacy, as real income value has declined across multiple professions, including nutrition and dietetics.

Multiple studies across different countries have reported dissatisfaction with promotion opportunities among dietitians, reflecting a consistent global concern in the profession. In our study, dietitians expressed slight dissatisfaction with promotion, mirroring trends observed elsewhere. Studies from South Africa, the United States, Australia and Canada all reported that promotion opportunities were among lowest-rated facets of job satisfaction, often linked to the limited senior roles in the profession, lack of leadership pathways and restricted upward mobility [11,44–46]. These findings collectively indicated that limited promotion prospects are a persistent issue in dietetics, regardless of region, and may be associated with overall job dissatisfaction and turnover rate [47].

When examining socio-demographic variables, job satisfaction in our sample was significantly associated with middle-aged groups (31–40 years), longer duration of practice (16–20 years), higher education levels, and higher income. These findings are consistent with studies conducted in South Africa [11], the United States [42,48], Brazil [49], and Germany [12].

Lower job satisfaction among younger dietitians may reflect early-career challenges documented in Lebanon and the region, including job insecurity, limited employment opportunities, low wages, and constrained career advancement pathways [50,51]. Evidence from international literature also suggests that newly graduated dietitians often experience difficulties transitioning into stable employment, underemployment, or roles that do not fully utilize their qualifications, which may negatively influence early-career job satisfaction [52–53].

Herzberg's Two-Factor Theory provides a useful framework for interpreting these patterns, suggesting that job satisfaction and dissatisfaction arise from two important factors; motivators that lead to satisfaction and hygiene factors that, if inadequate, contribute to dissatisfaction. Older and more experienced dietitians, who are more likely to hold higher positions and have greater motivators such as professional recognition, reported higher job satisfaction. Similarly, better paid and more highly educated dietitians may benefit from stronger hygiene factors such as job security and adequate compensation, thereby reducing dissatisfaction [54]. In this study, middle age was significantly linked to satisfaction with payment and nature of work. These associations may reflect developmental changes across the life course, as the relationships between income and life satisfaction tend to be stronger in midlife and weaker in younger and older ager, potentially moderating the influence of age on job satisfaction [55]. This is the result of the positive “spillover effect” whereby greater overall life satisfaction during midlife enhances job satisfaction [56]. No other significant associations were found between sociodemographic data and job satisfaction.

When job satisfaction was stratified according to employment location, dietitians practicing in Lebanon and abroad reported higher job satisfaction scores across most facets compared to those practicing in Lebanon only. Significant differences were observed particularly for payment, promotion opportunities, supervision, operating conditions, contingent rewards, co-workers, and nature of work, while no significant differences were found for fringe benefits, and communication scores were slightly higher among dietitians practicing in Lebanon only.

These findings are consistent with previous international literature suggesting that exposure to work environments outside the local context may be associated with higher satisfaction, potentially due to improved working conditions, clearer career pathways, and enhanced professional recognition [57]. In the Lebanese context, dietitians practicing abroad are more likely to benefit from higher income levels and stronger organizational support, which may collectively contribute to higher satisfaction across several job-related domains [58]. Interestingly, satisfaction with co-workers was also higher among dietitians practicing in Lebanon and abroad, possibly reflecting differences in workplace culture and team dynamics. Conversely, communication scores were marginally higher among dietitians practicing in Lebanon only, potentially reflecting closer interpersonal interactions within smaller or more familiar work environments [59]. Fringe benefits did not differ significantly, suggesting they may not be the primary drivers of satisfaction differences.

Regarding workload perception, no previous study has examined this aspect among Lebanese dietitians, and research on this topic remains limited globally. To the best of our knowledge, the only identified study assessing workload perception among dietitians was conducted in South Africa [11]. Other studies have addressed related topics such as burnout, which has been found to be high among dietitians and nutritionists, with heavy workloads making them more vulnerable to it [60]. In our study, the median workload score was 62.8%, indicating a slightly high perceived workload. Respondents reported their work as more mentally than physically demanding, consistently with the findings of van den Berg et al. [11], likely reflecting the predominance of clinical practice roles.

For work-related frustration, as conceptually defined by the NASA-TLX frustration subscale, refers to negative emotional states such as discouragement, irritation, stress and annoyance rather than qualitative participant feedback. Both studies reported slightly high frustration levels, with higher scores in Lebanon (60% vs 50%).

This discrepancy does not contradict prior literature suggesting associations between temporal demand and negative work-related emotions [61]; however, frustration appears to be influenced by multiple interacting factors, including resource availability, organizational support and broader socioeconomic conditions. In Lebanon, the economic context may represent an additional stressor, even when reported temporal demands were lower. Accordingly, frustration should be viewed as a multifactorial construct rather than the result of a single workload component, as supported by Sanclemente et al. [62].

When workload perception was stratified according to employment location, dietitians practicing in Lebanon only and those practicing in Lebanon and abroad reported comparable overall workload levels, with no statistically significant difference in total workload scores and both groups falling within the “slightly high” workload range. However, differences emerged at the level of specific workload components.

Dietitians practicing in Lebanon only reported significantly higher mental demand compared to those practicing in Lebanon and abroad, which may reflect greater cognitive strain associated with limited resources, staff shortages, and increased role burden within the local healthcare context. In contrast, frustration levels were significantly higher among dietitians practicing in Lebanon and abroad, suggesting that despite potentially better working conditions or compensation, challenges related to adaptation, dual practice responsibilities, or cross-cultural work environments may contribute to emotional strain. Temporal demand also differed significantly between the two groups, despite similar median scores, indicating distributional differences in perceived time pressure.

These findings are consistent with previous literature (e.g., Van den Berg et al. (11)) showing that workload perception is multidimensional and influenced not only by task volume but also by organizational context, resource availability, and psychosocial factors. Higher perceived workload, particularly in specific components such as mental demand and frustration, has been associated with lower job satisfaction, which aligns with the patterns observed in the present study [63]. Furthermore, according to Karasek’s Job Demand–Control model, differences in perceived workload across employment locations may reflect variations in autonomy, control, and support within different practice environments [64].

Despite being the first study addressing job satisfaction and workload perception among practicing dietitians in Lebanon, and one of the few to explore workload in this profession globally, this study had its limitations. The relatively small sample size (n = 138), the unequal distribution across some subgroups, particularly the public sector (8.7%), and dietitians with more than 20 years of experience, in addition to the Nabatieh area (1.4%) with no respondents from the southern district due to conflicts in the country during data collection, may have limited the statistical power of subgroup analyses and the precision of regression estimates. Consequently, findings related to these small groups should be interpreted cautiously. In addition, the use of convenience sampling instead of random sampling due to the lack of access to the national registry of dietitians constitutes another bias. The cross-sectional design of the study limits the ability to establish causal relationships between workload perception and job satisfaction. The observed associations should therefore be interpreted with caution, as the temporal direction between predictors and outcomes cannot be determined. Longitudinal studies would be required to better understand causal pathways. In addition, recruitment through social media platforms such as LinkedIn and Instagram may have introduced selection bias. Dietitians who are more active on professional or social media networks may have been more likely to encounter and respond to the survey. Dietitians who are less active online or who have limited access to these platforms may have been underrepresented. Consequently, the study sample may not fully reflect the characteristics of the entire population of practicing dietitians in Lebanon. Moreover, because the survey was distributed through social media platforms, the total number of dietitians who viewed the survey invitation was unknown; therefore, the response rate could not be calculated.

Finally, reliance on self-reported data which may be subject to bias due to dishonesty, misunderstanding, or external influences such as the prevailing conditions in Lebanon at the time of data collection might have temporarily affected dietitians’ responses.

Despite the above-mentioned limitations, the present study provides a valuable foundation for actions and future research. Policymakers, and healthcare administrators in collaboration with the Lebanese Order of Dietitians are encouraged to design strategies aimed at improving the dietitians’ workplace environment and overall career satisfaction. Addressing workload management in both sectors, offering competitive compensation, ensuring professional development opportunities, supporting career development and mentorship targeting young dietitians and incorporating stress management programs into the workplace can strengthen the profession and enhance the quality of care provided for patients. Furthermore, larger, and stratified random samples with broader geographic coverage could yield more representative results across both employment sectors and different Lebanese regions. Comparative studies with other countries in the region can shed the light on cultural factors influencing the job satisfaction and workload perception among dietitians, and qualitative research can provide richer insights into the lived experiences of dietitians.

The multivariable analyses further strengthen the results of the descriptive and stratified analyses by demonstrating that several sociodemographic and professional factors are independently associated with job satisfaction and workload perception among dietitians. In particular, income emerged as a key determinant of job satisfaction, and the significant interaction between income and practice location suggests that the higher satisfaction observed among dietitians practicing in Lebanon and abroad is largely driven by economic factors rather than practice location alone. In contrast, workload perception appeared to be influenced by a different set of factors, highlighting the multidimensional nature of workload and its partial independence from job satisfaction. These results underscore the importance of addressing both economic and organizational determinants when designing interventions to improve dietitians’ professional well-being.

## Supporting information

S1 File Questionnaire. (DOCX)

S1 Data Dataset. (XLSX)
