# Supplementary material for: Workload perception and job satisfaction among Lebanese practicing dietitians: A cross-sectional study by employment location
Source: PLoS One. 2026 Apr 7;21(4):e0346681. doi: 10.1371/journal.pone.0346681 (PMC13056163; doi:10.1371/journal.pone.0346681)
Supplement: S1 File — (DOCX) [file pone.0346681.s001.docx]

# Questionnaire

1. **Sociodemographic status**
2. What’s your date of birth? ___________
3. What’s your sexe?
   - Male
   - Female
4. What is your marital Status?
   - Married
   - Single
   - Widowed
   - Divorced
5. How many children do you have?
6. In which district do you live?
   - Beirut
   - Mount Lebanon
   - South

- Nabatieh
- Bekaa
- Baalbak-El Hermel
- Akkar
- North

1. **Employment and economic status**
2. What is your job style?
   - Part-time
   - Full-time
   - Free-lance
3. How do you classify your income status?
   - <100$
   - 100$-1000$
   - 1001-1500$
   - >1500$
   - No answer
4. **Education**
5. What is your educational level? (highest degree)
   - Holding a bachelor degree
   - Holding a master’s degree
   - Holding a PhD
6. Institution where the highest degree was obtained______________
7. Year graduated_____
8. **Professional experience**
9. Are you currently working in the Nutrition and Dietetics field?
   - No
   - Yes
10. What is the duration of practice of the profession?
    - <5 years
    - 5-10 years
    - 11-15 years
    - 16-20 years
    - >20 years
11. Country of practice
    - Lebanon
    - Lebanon and Abroad
    - Abroad
12. Areas of current employment (more than 1 could apply)
    - Cardiovascular diseases
    - Diabetes
    - Eating Disorders
    - Food allergies/intolerances
    - Food service management
    - Geriatrics
    - Gastrointestinal disorders
    - Critical care
    - Mental health
    - Oncology
    - Pediatrics
    - Pulmonary diseases
    - Renal diseases
    - Sports Nutrition
    - Other________________
13. Workplace (More than 1 could apply)
    - Private hospital
    - Governmental hospital
    - Private specialized center
    - Public specialized center
    - Private practice in clinic
    - Polyclinic
    - Nutrition services in sports centers, gyms and beauty clinics
    - Pharmaceutical company
    - Private university
    - Public university
    - Research
    - Food industry
    - Corporate
    - Public health services (with NGO)
    - Other_____
14. Workplace sector
    - Public
    - Private
15. **Job satisfaction survey**

|  | **JOB SATISFACTION SURVEY** | |  | |
| --- | --- | --- | --- | --- |
|  | PLEASE CIRCLE THE ONE NUMBER FOR EACH QUESTION THAT COMES CLOSEST TO REFLECTING YOUR OPINION  ABOUT IT. | | Disagree very much  Disagree moderately  Disagree slightly  Agree slightly  Agree moderately  Agree very much | |
| **Payment** | | |  | |
| 1 | I feel I am being paid a fair amount for the work I do. | | 1 2 3 4 5 6 | |
| 10 | Raises are too few and far between. | | 1 2 3 4 5 6 | |
| 19 | | I feel unappreciated by the organization when I think about what they pay me. | | 1 2 3 4 5 6 |
| 28 | | I feel satisfied with my chances for salary increases. | | 1 2 3 4 5 6 |
| **Promotion** | | |  | |
| 2 | There is really too little chance for promotion on my job. | | 1 2 3 4 5 6 | |
| 11 | Those who do well on the job stand a fair chance of being promoted. | | 1 2 3 4 5 6 | |
| 20 | | People get ahead as fast here as they do in other places. | | 1 2 3 4 5 6 |
| 33 | | I am satisfied with my chances for promotion. | | 1 2 3 4 5 6 |
| **Supervision** | | |  | |
| 3 | My supervisor is quite competent in doing his/her job. | | 1 2 3 4 5 6 | |
| 12 | My supervisor is unfair to me. | | 1 2 3 4 5 6 | |
| 21 | | My supervisor shows too little interest in the feelings of subordinates. | | 1 2 3 4 5 6 |
| 30 | | I like my supervisor. | | 1 2 3 4 5 6 |
| **Fringe benefit** | | |  | |
| 4 | I am not satisfied with the benefits I receive. | | 1 2 3 4 5 6 | |
| 13 | The benefits we receive are as good as most other organizations offer. | | 1 2 3 4 5 6 | |
| 22 | | The benefit package we have is equitable. | | 1 2 3 4 5 6 |
| 29 | | There are benefits we do not have which we should have. | | 1 2 3 4 5 6 |
| **Contingent rewards** | | |  | |
| 5 | When I do a good job, I receive the recognition for it that I should receive. | | 1 2 3 4 5 6 | |
| 14 | I do not feel that the work I do is appreciated. | | 1 2 3 4 5 6 | |
| 23 | | There are few rewards for those who work here. | | 1 2 3 4 5 6 |
| 32 | | I don't feel my efforts are rewarded the way they should be. | | 1 2 3 4 5 6 |
| **Operating conditions** | | |  | |
| 6 | Many of our rules and procedures make doing a good job difficult. | | 1 2 3 4 5 6 | |
| 15 | My efforts to do a good job are seldom blocked by red tape. | | 1 2 3 4 5 6 | |
| 24 | | I have too much to do at work. | | 1 2 3 4 5 6 |
| 31 | | I have too much paperwork. | | 1 2 3 4 5 6 |
| **Coworkers** | | |  | |
| 7 | I like the people I work with. | | 1 2 3 4 5 6 | |
| 16 | I find I have to work harder at my job because of the incompetence of people I work with. | | 1 2 3 4 5 6 | |
| 25 | | I enjoy my coworkers. | | 1 2 3 4 5 6 |
| 34 | | There is too much bickering and fighting at work. | | 1 2 3 4 5 6 |
| **Nature of work** | | |  | |
| 8 | I sometimes feel my job is meaningless. | | 1 2 3 4 5 6 | |
| 17 | I like doing the things I do at work. | | 1 2 3 4 5 6 | |
| 27 | | I feel a sense of pride in doing my job. | | 1 2 3 4 5 6 |
| 35 | | My job is enjoyable. | | 1 2 3 4 5 6 |
| **Communication** | | |  | |
| 9 | Communications seem good within this organization. | | 1 2 3 4 5 6 | |
| 18 | The goals of this organization are not clear to me. | | 1 2 3 4 5 6 | |
| 26 | I often feel that I do not know what is going on with the organization. | | 1 2 3 4 5 6 | |
| 36 | Work assignments are not fully explained. | | 1 2 3 4 5 6 | |

1. **Task-load index**

Scale the contribution of following factors to your daily work duties
